# The use of portable OSL and IRSL measurements of NaCl in low dose assessments following a radiological or nuclear emergency

**DOI:** 10.3389/fpubh.2022.969829

**Published:** 2022-08-30

**Authors:** Hamdan Alghamdi, David Sanderson, Lorna Carmichael, Alan Cresswell, L. Martin

**Affiliations:** Scottish Universities Environmental Research Centre, Glasgow, United Kingdom

**Keywords:** photo-stimulated luminescence (PSL), optically stimulated luminescence (OSL), NaCl, emergency dosimetry, retrospective dosimetry, portable OSL reader

## Abstract

During recovery phases following a nuclear or radiological incident analyses of doses received by members of the public and responders are often required. Several methods have been investigated for use at different timescales after the incident, including assessments based on measurements of materials present at the time of the incident. Common salt has previously been shown to have potential for retrospective dosimetry in the mGy dose range using laboratory instrumentation. This preliminary study investigates the use of portable instruments, with unprepared commercially sourced salt, in dose ranges below 100 μGy. Responses from pulsed IRSL and portable OSL instruments were compared. For OSL measurements, detection limits of 7 μGy have been demonstrated, with detection limits of 30–340 μGy for the other instruments investigated. Dose responses in the 0–500 μGy range were determined for the most sensitive systems, which show a linear response over this dose range with a non-zero intercept representing doses received from environmental sources since manufacture of the salt. For use as a dosimeter, methods of removing or accounting for inherited signals will be required in this low dose range. The results demonstrate that salt has considerable potential for use in retrospective dosimetry below 100 μGy, and that measurements can be conducted with portable OSL instruments.

## Introduction

Responses to potential nuclear or radiological incidents involve several phases. Initially there needs to be a rapid assessment of the nature of the incident, and priority given to protecting members of the public at greatest risk off-site. At this stage, real-time information is very important to guide decision makers. First responders would have to deal with civilian populations that may require decontamination, screening, medical treatment, housing, food, and water, as well as potential evacuation from affected areas. Responses may be impeded by loss of medical infrastructure, potentially large numbers of affected people and requirements to monitor potential contamination and doses received by first responders. Furthermore, a nuclear event evolves over time, with increasing numbers of victims, continued infrastructure loss, and the affected area's progression from fallout to deposition and redistribution leading to changing requirements for management and response (1). Thus, the management of radiation injuries or exposure will be critically dependent on the availability and application of appropriate measurement methods in a timely manner [e.g., (2)]. Compared to smaller incidents, where a small number of people are directly affected and even the most seriously injured can be helped by medical resources that are still in good shape, a large event may cause situations where the number of people affected could severely overwhelm any normal emergency medical resources that may be available. In these situations, information from appropriate measurements would let response managers triage casualties and decide how to best use limited available resources [e.g., (3)].

Instrumental measurements are capable of providing real-time information, both for response management and the protection of first responders. retrospective assessments may be conducted within a few days of the incident, and thus be available for emergency response and recovery, or several years later when the focus might also include scientific investigation of radiation effects and consequences.

Whereas, personal dosimeters and dose rate instruments are routinely provided to personnel assigned to nuclear emergency response organizations' rescue teams, provision of such devices to other first responders and members of the public is less common. during the early phases of emergency response it is highly unlikely that enough instrumental systems would be available to provide a comprehensive assessment of the extent of contamination and associated dose rates. moreover conventional personal dosimeters are not commonly used in the general population, resulting in limited timely information on the doses that individuals may have received. it is therefore often necessary to retrospectively estimate absorbed dose to make assessmentsof radiological impacts in an emergency situation and to provide accurate information to the general public.

The dose ranges of interest depend upon the assessment requirements. In the initial stages triage for people requiring immediate medical attention would require assessment of doses in excess of a few hundred msv, decisions on evacuation, sheltering, food and water restrictions, etc., would be based on dose rates with intervention levels typically of the order of tens of μGy hr^−1^ (4–8). Consideration of psychological effects and the mental health of potentially exposed individuals and communities may require assessments of dose at levels well below those for purely clinical requirements (9). Critical decisions need to be made on short timescales, and this requires dosimetric measurements and assessments immediately following the occurrence of an accidental or malicious use of radiation (10). In later stages of response it may be necessary to reconstruct the doses that individuals and communities received (11). retrospective analysis for recovery and scientific investigations may also require determination of smaller doses than those associated with early countermeasure decision making.

Several methods for dosimetric assessment in the absence of conventional personal dosimeters have been developed, following experience of nuclear weapons in Japan and nuclear accidents [e.g., (12)]. Instrumental measurements using portable gamma spectrometry systems and sample analysis can identify current radionuclide distributions, from which initial depositions can be estimated by modeling radionuclide decay and environmental re-distribution. A primary method of estimating doses received by individuals is through biodosimetry, the direct measurement of changes in human physiology associated with radiation exposure (13, 14). Advantages of biodosimetry include potential sensitivity to radiation damage, and the direct link between actual damage and clinical decision making. Disadvantages include potential lack of specificity as the same damage can be caused by other mechanisms, and involve invasive procedures with sample handling and analysis requiring specialist laboratories (13). Whole body monitoring and specific monitoring of individual body organs (e.g.,: the thyroid) allow measurement of internal contamination following inhalation and ingestion, which is an additional pathway for receiving radiation doses but beyond the scope of this work which focusses on external radiation exposure. Alternative methods which can complement modeled initial deposition is to estimate the dose absorbed by common objects and biological tissues collected from areas and individuals (14). This may be performed through the identification of radio-induced defects with physical techniques such as electron paramagnetic resonance (EPR), optically stimulated luminescence (OSL), and thermoluminescence (TL). Luminescence methods have been used in retrospective dosimetry to better understand past events and to study the impact of these (15, 16). These methods have been proposed to assist in emergency response but are not currently implemented within emergency response plans.

A study of OSL from electronic components of mobile phones and ID cards appears to have opened up the feasibility of dosimetry and dose reconstruction using the electronic components of gadgets of everyday use in the events of unforeseen situations of radiological accidents, including the event of a dirty bomb by terrorist groups (17). A variety of common household materials have been investigated for their potential use in osl dosimetry, including porcelain (18, 19), household salt [NaCl; e.g., (19, 20)], plastic cards, garments, shoes (21), salted crackers, almonds, pretzels, and potato chips (22).

Many of these techniques are suited to low to high dose regimes where identification of people who may develop medical conditions due to their exposure is required. There is, however, a requirement to confirm that individuals have not received a potentially harmful radiation dose, to provide reassurance to people, especially in situations where large numbers of people may have been affected. This needs a dosimetry system that can register ultra-low doses, and that could be widely available, or potentially deployed by pre-positioning suitable materials within areas at risk. Common table salt (NaCl) has been proposed as a retrospective dosimeter that would be widely present, it can be found in homes, workplaces, and restaurants all over the world. This means that collecting samples would be relatively simple, and sample preparation would be minimal.

Stoddard discovered in 1960 that light stimulation could cause luminescence in irradiated NaCl [as Reported by Geber-Bergstrand (23)]. Observations of TL and PSL from salt in the context of detecting irradiation of food have shown that this is a sensitive material (24–26). Bailey et al. (27) conducted studies of NaCl OSL, and concluded that OSL of NaCl may be a useful dating tool. Behring et al. (28) noted that sea salt has generally low background signals. Bernhardsson et al. (20) measured linear dose responses for five different salts using 470 nm blue LEDs to stimulate signals from 5 mg samples using a Risø DA-15 TL/OSL reader, with detection limits in the 0.1–1.0 mGy Range. Ekendahl and Judas (29) measured OSL from salt using riso OSL/TL readers, showing that salt is a sufficiently sensitive material for retrospective and accident dosimetry with a detection limit of 0.4 mGy. Christiansson (30) investigated the dose response for various salts also using riso readers, demonstrating favorable dosimetric features for low-dose (<100 mGy) applications, with a linear dose response in the range 1–100 mGy and detection limits as low as 0.2 mGy. Waldner (31) investigated the use of NaCl pellets for occupational dosimetry using OSL, with detection limits around 20 μGy, and also reviewed data for 102 different salts purchased in 47 countries showing variation in sensitivity particularly associated with iodine content.

TL studies of different salts have demonstrated peaks at ~100 and 260°C, with emissions at 590 nm (32–34). The 100°C peak has been shown to have a lifetime at 20°C of 7–14 h (33), whereas the 260°C peak has a lifetime of 4 ka (32, 34). Pulsed annealing OSL and IRSL experiments (33) identified OSL signals as originating from traps associated with both 100 and 260°C TL peaks, whereas the IRSL signals were identified as predominately originating from traps associated with the 100°C peak, and that there are no deeper traps in salt associated with either IRSL or OSL. Christiansson et al. (35) reported that OSL signals stimulated with blue diodes did not fade after 140 d storage, with a preheat of 10 s at 220°C. Low rates of fading (~5% over 2–4 weeks) have also been reported (19, 27, 29). More significant fading (35% after 7 days) was reported by Timar-Gabor and Trandafir (36) with a lower pre-heat at 150°C.

Demonstrations of the use of salt as a personal dosemeter in fukushima following the nuclear power plant accident, and in small salt containers in a village contaminated by the Chernobyl accident with salt packages left in kitchens for 4–5 months which measured doses down to 500 μGy (30, 35), have shown that radiation doses as low as 0.1–1.0 mGy region can be registered.

The work reported here extends these prior investigations to lower dose regions, using portable OSL measurement systems that could be deployed at or near the incident site to provide rapid measurement capability. This preliminary work assesses whether portable instruments can produce comparable or better sensitivity than laboratory based systems. Can these systems perform adequately for initial dose assessments under field conditions, thus removing the need to transport samples for analysis on laboratory instruments, and with sample throughput sufficient to provide significant quantities of quality data on emergency timescales? Can bulk produce be pre-calibrated prior to distribution, thus allowing initial dose assessments to be made without the need for individual regenerative calibration? For these questions, system performance will be evaluated through assessments of detection limits and dose response characteristics of replicated samples of commercially packed salt sachets. Further experiments will be needed to fully assess performance under potential application scenarios, but our initial questions concern sensitivity and reproducibility of measurements undertaken at ambient temperature using portable or field deployable instruments.

## Materials and methods

### Instrumentation

This work utilized three different portable instruments to measure luminescence signals from salt; a pulsed IRSL system developed for testing whether foods have been irradiated (25), a portable OSL system developed for luminescence measurements of sediments equipped with blue and IR diodes (37), and a variation on this with two different IR wavelength diodes for ultra-sensitive IRSL (38, 39).

### Photo-stimulated luminescence food instrument

The SUERC PSL instrument uses pulsed 880 nm IR diodes to stimulate luminescence, with a single photon counting photomultiplier to register the signal in an up-down counting mode where the dark count and any phosphorescence between pulses is subtracted from the luminescence during pulses (25). The instrument was developed to conduct screening measurements of foods that could have been subject to high dose radiation, following a standard method (40), but has also previously been used for retrospective dosimetry on bricks to 100 mGy detection limits (41). For this work, net counts from 60 s measurements were recorded.

### The SUERC portable OSL reader

The SUERC Portable OSL Reader (37) uses two sets of diodes for stimulation, which can be operated in continuous wave or pulsed modes, with a single photon counting photomultiplier. The system most commonly produced has 25 mW of 470 nm (blue) diodes and 90 mW 890 nm (IR) diodes, with a UG11 filter protecting the photomultiplier. A variation on this using 90 mW 890 nm and 90 mW 940 nm IR diodes with a 3 mm BG 39 filter has been developed to increase efficiency for IRSL measurements by an order of magnitude specifically for studies of young sediments (38) and also allowing investigation of IRSL response of feldspars to different stimulation wavelengths (39), and is also used in this work. For this work, the instruments were operated using a sequence described in Sanderson and Murphy (37) for rapid measurement of undifferentiated bulk polymineral samples, which is generally applicable to many samples. This uses a continuous wave mode with an interleaved series of measurements of 15 s dark count, two 30 s measurements with the longer wavelength diodes, 15 s dark count, two 30 s measurements with the shorter wavelength diodes, and 15 s dark count for each sample.

## Sample preparation

A box of 2,000 single serve salt sachets, all from a single batch, each of blue polyethylene containing 0.85–1.00 g >99.99% NaCl, was procured from a commercial retailer. Samples were prepared from randomly selected sachets for each experiment. All sample handling and measurement was conducted under subdued lighting.

For initial tests of response to beta irradiation, 30 samples were prepared on 30 mm diameter aluminum planchettes. These were sprayed with silicone grease as an adhesive before ~0.5 g of salt was spread evenly over the surface. Silicone grease is commonly used for this purpose because it does not affect the luminescence response of silicate minerals and other materials, including food stuffs (40). Ten of these were used to register the responses of each of the three luminescence instruments, before and after beta irradiation to a dose of 200 mGy. The initial measurements were made to determine the extent of any inherited luminescence from the as-prepared material. Samples were then exposed to beta-radiation at doses of 200 mGy at room temperature using a 0.62 GBq ^90^Sr source of surface area 4 cm^2^ mounted in a shielded irradiator with a working distance of 7.5 cm. The quartz equivalent dose rate in the sample position for this source had previously been determined (38) relative to a series of ^90^Sr sources in Elsec automatic irradiators and Risø readers in the luminescence dating laboratory at SUERC. These in turn had been calibrated relative to primary photon irradiations at the UKs National Physical Laboratory, and to secondary irradiations at SUERC. Following irradiation the samples were preheated for 30 min at a temperature of 50°C to remove unstable luminescence signals prior to re-measurement to establish the response to the 200 mGy dose.

To determine the dose response behavior in the microGray to sub-milliGray region 50 unopened sachets were exposed to gamma-ray doses of 20, 50, 100, 200, and 500 μGy These were irradiated in sets of 10 using a nominal 17 MBq ^60^Co source at a working distance of 20 cm. Ten sachets were retained as unirradiated controls. The sachets were weighed prior to use to confirm that they all contained the same mass of salt (gross weight 0.99–1.00 g). The dose rate for the ^60^Co source had been estimated from the source activity, sample to source distance and mean gamma ray energies. This was verified in March 2021, using Fontainebleau quartz grains placed in 5 mm diameter quartz tubes within a 1 mm thick aluminum tube to develop charged particle equilibrium and gamma irradiated to estimated doses of 50 and 100 mGy (42). The powder was dispensed on disc for measurement on a Risø DA-15 OSL/TL reader, along with additional sets for control beta irradiations and a set of blanks. OSL analysis and renormalization using the ^90^Sr source in the Risø reader was conducted and an updated ^60^Co dose rate at this working distance was estimated. Decay corrections were applied to the ^60^Co dose rate determined, using the half life of 5.27 y. The full contents of each sachet were emptied into 50 mm diameter petri dishes to give an evenly distributed layer on the bottom to present to the instruments (10 sachets at each dose for each instrument). For the dose response experiments to photon irradiation in the 20–500μGy regions samples were stored under dark conditions at room temperature for 10 days to allow any unstable signal components to decay before measurement. Prior studies, as outlined above, indicate that signals from salt do not fade significantly after a few days if pre-heating is used to remove signals associated with shallow traps. The 10d period was selected pragmatically, in order to ensure that the small differences in storage time between irradiation and measurement were small compared with this post-irradiation delay. No further preheating was undertaken at this stage.

## Experimental measurements

### Comparison of instruments

The experiment used beta irradiated samples on 3 cm planchettes which had received nominal 200 mGy doses. Ten samples were measured in each of the three instruments, with both Portable OSL instruments using two wavelengths giving five sets of measurements. Each measurement sequence started and ended with empty chamber backgrounds, and reference materials to confirm functionality of the instruments.

The counts for each measurement from the 200 mGy irradiated samples were used to determine a sensitivity (photon counts per Gray, c Gy^−1^) from the 0.5 g of salt for each instrument and light source. The measurements prior to irradiation showed a residual signal that was small compared to the signals after receiving the 200 mGy dose. Measurements of salt samples that had been exposed to artificial daylight for 1 week showed luminescence signals significantly above the empty chamber, especially with 890 and 470 nm stimulation, and it is concluded that obtaining a salt sample with zero luminescence to act as a blank is impractical for this evaluation of the systems. For the purposes of estimation of minimum detectable doses in this study we consider that the empty chamber blank is satisfactory. The empty chamber background measurements produced consistent values, and minimum detection limits (MDL) were determined as the mean of these counts plus three times their standard deviation. These were then converted to dose using the sensitivity calculation, assuming a linear dose response over this range which is expected from the prior studies of luminescence from salt, giving a minimum detectable dose (MDD) in mGy.

### Dose response

The experiment used the salt sachets which had been gamma irradiated to low dose (0–500 μGy) to develop a dose response curve over this dose range, which incorporates the range of interest for public reassurance. Ten samples at each dose, including unirradiated controls, were measured initially using the instrument with the lowest detection limit, the Portable Reader operating with 470 nm blue LEDs. Each sachet was emptied into a 50 mm diameter petri dish for measurement, and the net photon counts recorded. The experiment was repeated later using the food instrument and the IRSL system at 890 nm.

## Results

### Backgrounds, sensitivities and detection limits

The count rates and standard deviations for measurements of the background and the salt samples irradiated to 200 mGy, for all five sets of measurements, are given in Table 1. The highest photon counts, from the 200 mGy dose, and lowest detection limit were given by the portable OSL instrument using blue LEDs (2,303.3 ± 807.0 kc with a MDD of 6.7 μGy). All other systems gave lower counts and higher detection limits. The sensitive IR portable OSL system gave 605.9 ± 60.9 kc at 890 nm from 200 mGy (compared with 43.3 ± 1.7 kc for the same wavelength in the less sensitive system) and a MDD of 31 μGy. Note that the measurement sequence had already depleted some signal from the samples prior to the 940 nm measurements on this system, which may explain the lower sensitivity recorded. The PPSL instrument using IR stimulation in up-down counting mode gave counts of 279.2 ± 71.2 kc from 200 mGy, and a MDD of 88 μGy.

**Table 1:** Photon counts and standard deviation for the background and 200 mGy irradiated salt samples for five sets of measurements on three instruments, with calculated sensitivities and detection limits.

	**Instrument and wavelength**				
	**PPSL**	**Portable OSL 1**		**Portable OSL 2**	
		**IR (890 nm)**	**IR (940 nm)**	**IR (890 nm)**	**OSL (470 nm)**
Background (empty chamber)	20 ± 34	−10 ± 32	−4 ± 31	45 ± 25	−61 ± 26
MDL (counts)	123	95	94	74	78
Pre-irradiation counts	113 ± 72	−4 ± 73	−146 ± 89	−27 ± 128	673 ± 160
200 mGy counts	279,200 ± 71,200	605,900 ± 60,900	65,500 ± 21,800	43,300 ± 1,700	230,3300 ± 80,700
Sensitivity (counts mGy^−1^)	1,400 ± 240	3,030 ± 300	330 ± 110	220 ± 10	11,520 ± 400
MDD (mGy)	0.088	0.031	0.286	0.340	0.0067

All the instruments used here can have a useful role in dosimetric measurements using salt. The detection limits for the PPSL and the IR only portable system using 0.5 g samples are lower than previously reported for measurements using Riso readers which reported detection limits above 100 μGy using aliquots with mg quantities of salt (20, 29). However, the blue OSL system is significantly more sensitive with lower detection limits.

### Dose response

**Figure 1:**
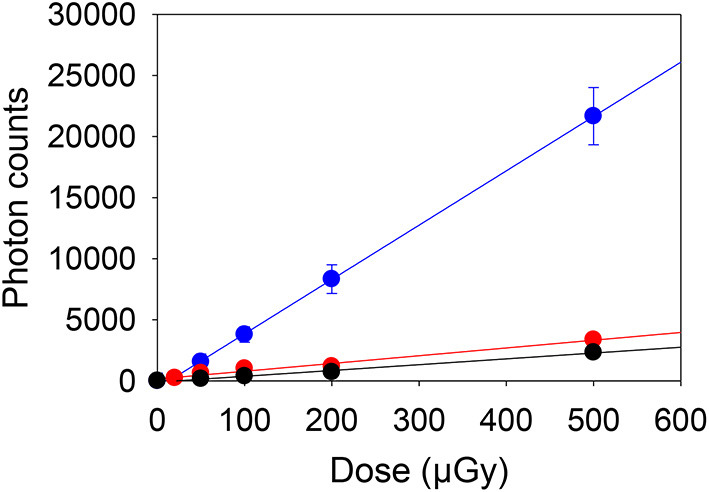
Dose response from gamma irradiated samples measured using a blue OSL portable reader (blue), 890 nm IRSL (red) and the pulsed PSL (black) instruments, after subtraction of the signals from the salt which did not receive additional doses.

The gamma irradiated materials were used to construct dose response curves for salt irradiated to low dose (0–500 μGy). The mean net photon counts and standard deviation recorded from 10 samples of salt at each dose are given in Table 2, with the mean counts, after subtracting the mean counts for the 0 μGy samples, plotted in Figure 1. For the blue OSL signals the data in Figure 1 are well-described by a linear regression (slope 44.5 ± 0.9 c μGy^−1^. The 0 μGy signal would correspond to a residual dose of 66.1 ± 4.4 μGy. Dose response curves for the other instruments and wavelengths (Figure 1) also show linear regressions with smaller slopes (6.4 ± 0.5 and 4.8 ± 0.2 c μGy^−1^ for the 890 nm IRSL and pulsed PSL respectively) reflecting the different sensitivities (Table 1). The signals without an additional dose are simply explained as a result of prior exposure of the salt to environmental doses arising from naturally occurring radionuclides and cosmic rays. For the initial measurements using blue OSL, this corresponded to a dose of 66.1 ± 4.4 μGy. The subsequent measurements, 105d later, using IR (890 nm) stimulation gave a dose of 213 ± 23 μGy, the difference corresponding to a dose rate of 0.058 ± 0.001 μGy h^−1^. The gamma dose rate in the cupboard where the salt is stored was measured as 0.063 ± 0.002 μGy h^−1^ using a 2 × 2” NaI spectrometer, which is entirely consistent with the values observed.

**Table 2:** Mean and standard deviation of photon counts recorded using the SUERC portable OSL reader at 470 nm, the IRSL instrument at 890 nm and the pulsed PSL instrument, from sets of ten individual sachets of salt exposed to gamma doses from 0–500 μGy.

	**Portable OSL 470 nm**		**Portable IRSL 890 nm**		**Pulsed PSL 880 nm**	
**Dose (μGy)**	**Counts**	**CV (%)**	**Counts**	**CV (%)**	**Counts**	**CV (%)**
0	3,534 ± 453	12.8	1,204 ± 313	26.0	1,324 ± 102	7.7
20	3,307 ± 689	20.8	1,443 ± 300	20.8	1,256 ± 150	12.0
50	5,091 ± 460	9.0	1,843 ± 607	32.9	1,487 ± 122	8.2
100	7,323 ± 444	6.1	2,214 ± 202	9.1	1,701 ± 143	8.4
200	11,860 ± 1,086	9.2	2,388 ± 655	27.5	2,038 ± 237	11.7
500	25,202 ± 2,303	9.1	4,565 ± 687	15.1	3,649 ± 385	10.6

## Discussion and conclusions

This preliminary investigation, in line with previous work, has demonstrated that salt shows highly promising dosimetric properties for retrospective dosimetry and rapid dose assessment in an emergency situation. Furthermore, the work reported here has shown that portable instruments can make low dose determinations, significantly below 100 μGy, more quickly and conveniently than using laboratory based instruments. Using bulk prepacked materials the results from sets of 10 sachets are reproduced to high precision across a series of doses, suggesting that initial dose assessments can be made for such material without individual re-calibration of each dosimeter. For readout using the blue LEDs in the SUERC Portable OSL Reader minimum detection limits of 7 μGy have been determined from single ~1 g salt sachets. A linear absorbed dose-response relationship was observed in the 0–500 μGy range. A non-zero intercept corresponding to an initial dose of 60–70 μGy was noted, and is likely to correspond to an environmental dose given to the salt sachets in the period since manufacture. The other instruments investigated would also be suitable for such applications, though with slightly higher detection limits for the PPSL instrument developed for food analyses and the higher sensitivity IRSL version of the portable reader.

Thus, such instruments would be capable of measurements of doses <100 μGy, and can be used under battery operation in the field or nearby location. This would allow the deployment of multiple systems, and measurement of samples without the need to transport them to remote laboratories. Common reference materials are already supplied with the instruments, these would be able to account for variations in stimulation power or photomultiplier response between different instruments. Pre-calibration of salt samples from single batches, or across multiple batches should they be sufficiently similar in luminescence characteristics, would allow measurements without requiring additional calibration doses in the field.

The measurement protocol used here involved 60 s measurements per aliquot, allowing measurements of 30–40 aliquots per hour on a single instrument. For these OSL measurements, 90% of the signal was generated within 15 s so shorter measurement times per sample, and more aliquots per hour, would be possible without significant loss of sensitivity. For this work, 10 aliquots were used per sample, however the coefficients of variation at <10% would permit lower levels of replication, and hence more samples per hour. Further investigation of the interplay between measurement sequence, sample throughput, sensitivity and the influence of post irradiation delays on signal response would be useful.

Prior studies have indicated that the OSL signal, after allowing for decay of the less stable components, has a long lifetime at 20°C (32, 34) suitable for retrospective dosimetry. Further studies would be needed to assess stability of this signal at elevated environmental temperatures, and to develop measurement protocols to either allow less stable components to decay prior to measurement (which could include pre-heating) or to account for these signals.

The observation of an initial signal prior to artificial irradiation indicates that salt is sensitive enough for dosimetry at natural environmental dose rates. For low-dose retrospective dosimetry methods to either remove or account for this initial signal would need to be investigated.

The samples analyzed here showed very small dispersion in the response to radiation doses, thus a dose response curve and initial signal can be determined from a small subsample of the batch prior to deployment, and applied to all of the sachets. If other salt samples show the same responses to known doses then a universal calibration may be possible. As noted by Waldner (31), investigations of the properties of each specific salt is warranted prior to use in dosimetric applications, this would identify whether a general response curve is applicable or whether a curve specific to that salt would be needed. Further work is needed to evaluate the responses of different sources of salt, and to consider possible modes of deployment of bulk material of this sort, and the potential for initial rapid estimates of dose to be made under emergency conditions using pre-calibrated materials.

The practicality of using salt as a retrospective dosimeter in response to nuclear or radiological emergencies would also need to manage deployment and collection of salt samples without light exposure or hydration. Christiansson et al. (35) have demonstrated that white plastic containers or paper sachets do not sufficiently protect the sample from light exposure, whereas cardboard boxes and black plastic containers do.

This study has conclusively demonstrated that dosimetry using common salt with a portable OSL reader is a rapid and effective means of determining dose below 100 μGy. Some further work on defining precise details for the use of such systems in nuclear or radiological emergency response is still required.

## Data availability statement

The raw data supporting the conclusions of this article will be made available by the authors, without undue reservation.

## Author contributions

The experimental work was conducted by HA in consultation with DCWS, supported by LC, AC, and LM. The manuscript was prepared by HA with editorial contributions from all authors. All authors contributed to the article and approved the submitted version.

## Funding

The work was conducted at SUERC within the PhD programme of HA, supported by a scholarship from the Government of the Kingdom of Saudi Arabia. Gamma source calibration was conducted by LM, with support from Royal Society Newton International Fellowship (NIF/R1/192806).

## Conflict of interest

The authors declare that the research was conducted in the absence of any commercial or financial relationships that could be construed as a potential conflict of interest.

## Publisher's note

All claims expressed in this article are solely those of the authors and do not necessarily represent those of their affiliated organizations, or those of the publisher, the editors and the reviewers. Any product that may be evaluated in this article, or claim that may be made by its manufacturer, is not guaranteed or endorsed by the publisher.
